# Cost-effectiveness analysis of advanced radiotherapy techniques for post-mastectomy breast cancer patients

**DOI:** 10.1186/s12962-020-00222-y

**Published:** 2020-08-03

**Authors:** Yibo Xie, Beibei Guo, Rui Zhang

**Affiliations:** 1 Medical Physics Program, Department of Physics and Astronomy, Louisiana State University, Baton Rouge, LA USA; 2 Department of Experimental Statistics, Louisiana State University, Baton Rouge, LA USA; 3 Department of Radiation Oncology, Mary Bird Perkins Cancer Center, Baton Rouge, LA USA

**Keywords:** Cost effectiveness analysis, Post-mastectomy, Breast cancer, Radiotherapy

## Abstract

**Background:**

Prior cost-effectiveness studies of post-mastectomy radiotherapy (PMRT) only compared conventional radiotherapy versus no radiotherapy and only considered tumor control. The goal of this study was to perform cost-effectiveness analyses of standard of care (SOC) and advanced PMRT techniques including intensity-modulated radiotherapy (IMRT), standard volumetric modulated arc therapy (STD-VMAT), non-coplanar VMAT (NC-VMAT), multiple arc VMAT (MA-VMAT), Tomotherapy (TOMO), mixed beam therapy (MIXED), and intensity-modulated proton therapy (IMPT).

**Methods:**

Using a Markov model, we estimated the cost-effectiveness of various techniques over 15 years. A cohort of women (55-year-old) was simulated in the model, and radiogenic side effects were considered. Transition probabilities, utilities, and costs for each health state were obtained from literature and Medicare data. Model outcomes include quality-adjusted life-years (QALYs) and incremental cost-effectiveness ratio (ICER).

**Results:**

For the patient cohort, STD-VMAT has an ICER of $32,617/QALY relative to SOC; TOMO is dominated by STD-VMAT; IMRT has an ICER of $19,081/QALY relative to STD-VMAT; NC-VMAT, MA-VMAT, MIXED are dominated by IMRT; IMPT has an ICER of $151,741/QALY relative to IMRT. One-way analysis shows that the probability of cardiac toxicity has the most significant impact on the model outcomes. The probability sensitivity analyses show that all advanced PMRT techniques are more cost-effective than SOC at a willingness-to-pay (WTP) threshold of $100,000/QALY, while almost none of the advanced techniques is more cost-effective than SOC at a WTP threshold of $50,000/QALY.

**Conclusion:**

Advanced PMRT techniques are more cost-effective for breast cancer patients at a WTP threshold of $100,000/QALY, and IMRT might be a cost-effective option for PMRT patients.

## Background

About 1 in 8 US women will develop invasive breast cancer over the course of her lifetime, and the number of women being diagnosed continues to increase (seer.cancer.gov). A mastectomy is highly recommended for patients with locally advanced primary breast cancer and extensive lymph node involvement, and post-mastectomy radiotherapy (PMRT) has been shown to improve the overall survival for invasive breast cancer patients by reducing the risk of tumor recurrence and cancer mortality [1].

The current standard of care (SOC) PMRT technique in the US is using conventional parallel-opposed tangent photon fields to treat the lateral chest wall plus oblique electron fields; for patients with advanced disease, supraclavicular and axillary nodes are treated with additional photon fields [2]. In the past decades, many advanced technologies had been used for PMRT and shown promising results, such as intensity-modulated radiation therapy (IMRT) [3], standard volumetric modulated arc therapy (STD-VMAT) [4], non-coplanar VMAT (NC-VMAT) [5], multiple arc VMAT (MA-VMAT) [5], Tomotherapy (TOMO) [4], bolus electron conformal therapy (BECT) [6], BECT mixed with IMRT and VMAT (MIXED) [7], and proton therapy [8], each with different degrees of sophistication and cost. Target coverage provided by most of these technologies is comparable, while the dose to surrounding normal tissues varies greatly. The more advanced technique have the potential to improve treatment quality by constraining therapeutic dose to radiosensitive organs, but they also have drawbacks like increased low-dose volume which could increase the risk of developing side effects [9, 10]. Long-term breast cancer survivors could develop chronic treatment-related morbidity and even mortality after PMRT including cardiac toxicities and secondary cancers etc. [11–16], which may significantly decrease their quality of life, e.g. it has been reported one radiogenic second cancer occurred in every 200 women treated with radiotherapy [16].

Among all cancer sites, expenditures for female breast cancer remain the highest and will continue to rise to $20.5 billion by 2020 [17]. It is controversial whether the additional costs of advanced radiotherapy techniques are justified by the potential advantages. Prior cost-effectiveness studies of PMRT only compared conventional radiotherapy versus no radiotherapy and only considered tumor control [18, 19]. The cost-effectiveness of newer techniques including costs of treating late radiogenic side effects has not yet been examined. Given the prevalence of breast cancer and continued growth of health care costs, results from such an analysis will have a positive impact and can help choose the most cost-effective PMRT technique for the patients.

The goal of this study was to perform cost-effectiveness analyses of various PMRT techniques including conventional SOC, fixed-beam IMRT, STD-VMAT, NC-VMAT, MA-VMAT, TOMO, MIXED, and intensity-modulated proton therapy (IMPT). Besides tumor coverage, late side effects (cardiac toxicity and secondary cancers) after PMRT were also included in the analyses.

## Methods

### Decision model

**Fig. 1:**
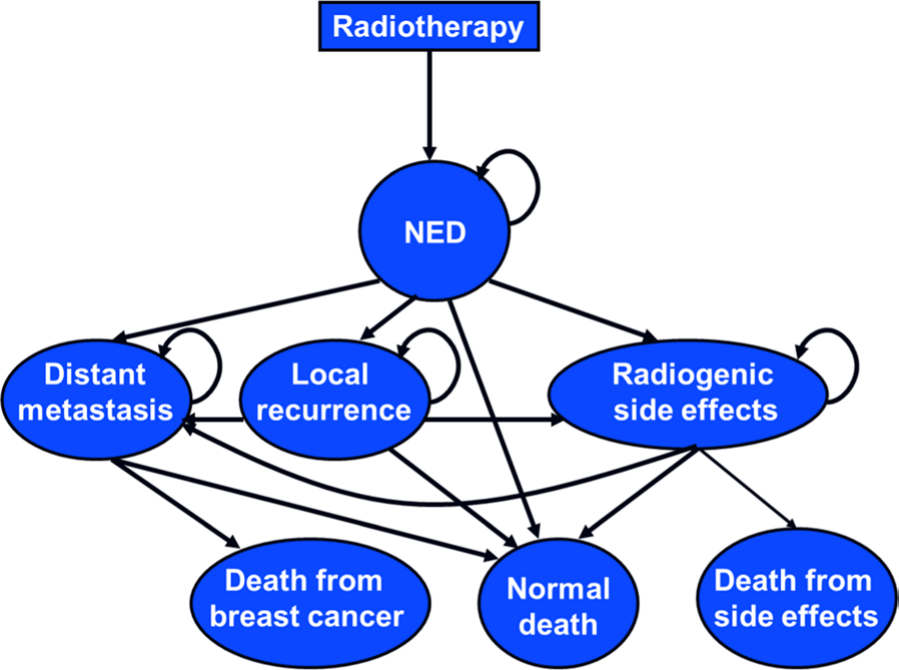
Overview of the Markov model. *NED* no evidence of disease

A Markov model (Fig. 1) was built using an in-house code to simulate the clinical history of one hypothetical cohort of women who received PMRT with a prescribed dose of 50 Gy in 25 fractions. The cohort consisted a population of 55-year-old postmenopausal women with Stage III breast cancer (node positive with tumor diameter smaller than 2 cm) after mastectomy, and the planning target volume (PTV) for these patients included the left chest wall, left supraclavicular and axillary areas, and internal mammary chain area. Markov simulation allowed these patients to transition between different health states, including no evidence of disease (NED), distant metastasis, local recurrence, late radiogenic side effects, and death, in a fixed increment of time (1 year). The primary endpoints of this study included quality-adjusted life years (QALYs) from a payer perspective over a 15-year horizon. Treatment strategies associated with lower costs and higher QALY were considered dominant. Incremental cost-effectiveness ratios (ICERs), which was defined as the incremental cost divided by the incremental QALY gained, were calculated in scenarios where there was no dominant strategy. We will determine whether a PMRT modality is cost-effective by comparing ICER with common willingness-to-pay (WTP) thresholds of $50,000/QALY and $100,000/QALY [20, 21].

### Model data input

The transition probabilities for SOC PMRT were taken from literature [12, 18, 22–27]. Table 1 shows transition probabilities for 55-year-old PMRT patients receiving SOC PMRT: each baseline value of transition probability was summed over years been studied and the value divided by the number of years was used in the Markov model. Table 1 also shows the utilities values for each health state in the Markov model.

**Table 1:** Transition probability and utility for the 55-year-old cohort

Parameters	Years	Value (%) (range)	References
Probability			
Local Recurrence	0–5	6.5	[22]
	6–10	1.84	
	11–15	0.325	
NED to metastasis	0–5	18.1	[23]
	6–10	9.2	
	11–15	5.68	
Metastasis to death	0–5	25.9	[18]
	6–15	15.6	
Normal death	56–60 (age)	3.2	[30]
	61–65 (age)	4.7	
	66–70 (age)	5.8	
Death due to lung toxicity	11–15	0.49	[25]
Death due to heart toxicity	11–15	2.6	[25]
Death due to CL breast toxicity	11–15	21.7	[22]
SOC PMRT cardiac toxicity	11–15	5.84	[12]
SOC PMRT CL breast cancer	6–15	1.0	[26]
SOC PMRT lung cancer	11–15	4.4	[27]
Utility			
Cardiac toxicity	11–15	0.85 (0.8–0.9)	[35]
CL breast cancer	11–15	0.803 (0.708–0.816)	[43]
Lung cancer	11–15	0.72 (0.57–0.87)	[43, 44]
Recurrence	0–15	0.85	[18]
NED to metastasis	0–15	0.62	[18]

NED: no evidence of. CL breast: contralateral breast

For advanced PMRT techniques, the transition probabilities were largely lacking in the literature. In this study, probabilities of tumor coverage including local recurrence and metastasis after advanced PMRT were assumed to be the same as those after SOC PMRT, while probabilities of radiogenic side effects after advanced PMRT were calculated based on normal tissue complication probability (NTCP) [28], lifetime attributable risk (LAR) of second cancers [29, 30] and risk of coronary events (RCE) [12, 31] models for a 55-year-old cohort as we previously reported [5, 8] (Table 2). We assumed that all lung and cardiac events start from year 11 after radiotherapy [12, 32], while contralateral breast events start from year 6 after radiotherapy [26].

**Table 2:** Calculated probabilities of developing radiogenic side effects for the 55-year-old cohort from previous studies [5, 8]

Side effect	Probability (%)	Range (%)
IMRT cardiac toxicity	0.95	0.05–2.58
IMRT CL breast cancer	0.04	0.02–0.06
IMRT lung cancer	0.57	0.08–0.84
STD-VMAT cardiac toxicity	0.97	0.05–2.54
STD-VMAT CL breast cancer	0.11	0.06–0.23
STD-VMAT lung cancer	0.63	0.08–0.95
NC-VMAT cardiac toxicity	0.89	0.05–2.34
NC-VMAT CL breast cancer	0.08	0.02–0.20
NC-VMAT lung cancer	0.54	0.07–0.89
MA-VMAT cardiac toxicity	0.89	0.05–2.31
MA-VMAT CL breast cancer	0.09	0.04–0.20
MA-VMAT lung cancer	0.55	0.07–0.92
TOMO cardiac toxicity	0.98	0.07–2.39
TOMO CL breast cancer	0.10	0.06–0.22
TOMO lung cancer	0.72	0.11–1.18
MIXED cardiac toxicity	0.86	0.05–2.22
MIXED CL breast cancer	0.07	0.03–0.15
MIXED lung cancer	0.63	0.08–1.01
IMPT cardiac toxicity	0.40	0.04–0.84
IMPT CL breast cancer	0.003	0.00–0.007
IMPT lung cancer	0.22	0.03–0.60

*CL breast* contralateral breast

The annual mortality rates due to breast cancer were derived from Early Breast Cancer Trialists’ Collaborative Group (EBCTCG) [1]. The normal death rates were based on the United States life tables [30]. The death from the radiogenic side effects was mainly caused by cardiac toxicity and second cancers [25].

Table 3 shows the costs for PMRT patients using different technologies from payer perspective and these costs were based on local Medicare charges. Costs of treating late effects were also included in Table 3. All costs and utilities were discounted at 3% per year as recommended by US Panel on Cost-Effectiveness in Health and Medicine [33].

**Table 3:** Treatment costs

Treatment	Cost (range)	References
PMRT (SOC)	$12,140	Based on medicare charge
PMRT (VMAT/TOMO/IMRT)	$17,438	Based on medicare charge
PMRT (MIXED)	$19,715	Based on medicare charge
PMRT (IMPT)	$33,547	Based on medicare charge
Local recurrence	$20,879	[45]
Metastasis	$13,627	[45]
Cardiac toxicity	$11,570 (8165–14,975)	[35, 46]
CL breast cancer	$14,494 (13,295–15,693)	[47]
Lung cancer	$20,577 (17,837–23,317)	[48–50]

CL breast: contralateral breast

### Model calibration and validation

CancerMath is the latest web-based breast cancer prognostic tool that can predict mortality rate in each year for the first 15 years after the current SOC treatment, and the external validity of our model was assessed by comparing 15-year overall survival and breast cancer mortality of patients who received SOC PMRT with the predicted results from CancerMath.

### Sensitivity analyses

We performed a series of one-way sensitivity analyses to determine the variability in the ICER as a function of the probabilities, utilities, and treatment costs of contralateral breast cancer, lung cancer and heart toxicities for seven advanced PMRT techniques versus SOC PMRT.

We also performed probability sensitivity analyses (PSA). The probabilities, utilities and costs were varied simultaneously across their distributions using a second-order Monte Carlo simulation. Transition probabilities and utilities were modeled using a beta-distribution and cost was modeled using a gamma distribution as recommended in the literature [34]. The cost-effectiveness acceptability was calculated based on the result of 100,000 simulations for each PMRT technique at different WTP thresholds.

## Results

For 55-year-old women with breast cancer, our model predicted a 15-year overall survival rate of 69.7% and breast cancer mortality rate of 19.2%, whereas CancerMath estimated an overall survival rate of 70.5% and breast cancer mortality rate of 18.0%. These comparisons suggest that our model’s predictions are similar to real clinical outcomes.

Treatment cost, QALY, and ICER values for all seven advanced PMRT techniques are shown in Table 4 and are listed in order of increasing cost. ICRE values are calculated for each technique relative to the adjacent cost-effective technique, e.g. ICER of STD-VMAT is relative to SOC, ICER of TOMO is relative to STD-VMAT, while ICER of IMRT is relative to STD-VMAT because TOMO is dominated by STD-VMAT and therefore is not considered cost-effective. Additional file 1: Figure S1 also illustrates the comparisons among all PMRT techniques. Additional file 1: Figure S2 shows one-way sensitivity analyses, which indicate model outcomes are most significantly impacted by the probability of developing cardiac toxicity.

**Table 4:** Cost, quality-adjusted life-years (QALY), and incremental cost-effectiveness ratio (ICER) values

	Cost ($)	QALY	ICER ($/QALY, relative to the adjacent cost-effective technique)
SOC	15,352	9.214	–
STD-VMAT	19,131	9.33	32,617
TOMO	20,267	9.314	Dominated by STD-VMAT
IMRT	20,543	9.404	19,081
MA-VMAT	20,617	9.365	Dominated by IMRT
NC-VMAT	20,633	9.402	Dominated by IMRT
MIXED	22,985	9.385	Dominated by IMRT
IMPT	38,145	9.52	151,741

WTP acceptability curves for seven techniques are shown in Additional file 1: Figure S3. Table 5 depicts PSA results which show the probability of being more cost-effective than SOC for the advanced PMRT techniques. None of the advanced techniques has an over 31% probability of being more cost-effective at a WTP of $50,000/QALY, while all advanced techniques are more cost-effective at a WTP of $100,000/QALY.

**Table 5:** Probability of being more cost-effective than SOC for advanced PMRT techniques

WTP ($/QALY)	IMRT (%)	STD-VMAT (%)	NC-VMAT (%)	MA-VMAT (%)	TOMO (%)	MIXED (%)	IMPT (%)
50,000	30.7	15.6	6.1	1.8	0.6	2.4	0.0
100,000	99.8	96.3	99.0	97.4	88	97.2	99.9

## Discussion

To the best of our knowledge, this study evaluated the cost-effectiveness of seven advanced PMRT techniques compared with SOC for the first time. Both tumor coverage and radiogenic late effects were considered in our model. The uncertainty of probabilities, utilities and treatment costs of radiogenic late effects were analyzed using one-way analysis and PSA.

We found most of the advanced techniques would be more cost-effective than SOC PMRT at a WTP of $100,000/QALY. IMRT exhibits the lowest ICER relative to SOC ($27,310/QALY) among all techniques for the patient cohort, which is possibly because fixed beam IMRT can significantly reduce contralateral breast doses and lower the probability of developing secondary cancer in contralateral breast. IMPT has the highest ICER relative to SOC ($74,564/QALY), which is mainly due to the high cost of the initial treatment (Table 3).

An important strength of our study is the consideration of radiogenic side effects, which was rarely done in the previous cost studies. Given the favorable oncologic outcomes, it is increasingly paramount to minimize radiogenic side effects and improve survivorship for these patients. Lundkvist et al. [35] reported that proton therapy is more cost-effective than conventional radiation therapy if breast cancer patients with high-risk of cardiac disease are treated. Mailhot et al. [36] also reported the heart dose and cardiac risk factor are the key factors for cost-effective allocation of proton therapy for breast cancer. Although these two studies were not specifically designed for PMRT, their findings are consistent with ours in that our study also shows the model outcomes are most sensitive to the probability of developing cardiac toxicity. This may due to the fact the risk value of cardiac toxicity is higher than the risk value of lung cancer or contralateral breast cancer for breast cancer patients [12, 26, 27].

Our study has broad implications. It will provide a quantitative comparison of cost-effectiveness among contemporary PMRT strategies, and enhance the base of evidence upon which clinical decisions can be made, e.g. it has been highly controversial if the additional cost of proton therapy is justified by the potential advantages, and our study shows proton therapy is only cost-effective for PMRT patients at a WTP threshold of 100,000 $/QALY although it confers the lowest risks of radiogenic side effects among all techniques [5, 8]. It may lower the national cost of breast cancer care, and even a small progress in this direction can help relieve patient’s burden and national healthcare pressure. The scarce healthcare resources can be saved and used in other aspects of patient care. It can inspire healthcare providers to replace the current SOC with ones that are more effective and less toxic, and also inspire stakeholders to make stronger cost-containment measures and integrate efficacy and cost-effectiveness into clinical trials in the US, which can potentially bring in enormous savings of time, money and human resources.

There are several limitations of this study. First, there is a lack of clinical information on late toxicities from advanced PMRT techniques, and the well-defined risk models were used to estimate those risk values in our study. We expect there will be certain degree of discrepancies in absolute outcome values between our study and future clinical data, but we do not expect dramatic differences in relative values, i.e. the rank of alternative techniques. By taking into account possible uncertainties, we are confident that our model is robust. Long-term prospective studies are needed to better explore the probability of the late effects and their impact on the cost-effectiveness of various radiotherapy modalities. Second, we did not include the uncertainties of local recurrence and distance metastasis in PSA analyses since we assume that all advanced techniques have the same tumor control probability as conventional SOC. Although there is no clinical evidence currently, it is possible advanced techniques like proton therapy will bring benefits on the survival from the primary cancer, and the cost-effectiveness of advanced techniques will improve. Third, we conducted the study only from a payer perspective, while the US Panel on Cost-Effectiveness in Health and Medicine recommended both patient and societal analyses should be presented [37]. The significant capital cost associated with the advanced technique like proton therapy will lower its cost-effectiveness even further. However, as Sher et al. [38] pointed out, the cost-effectiveness analysis from the payer perspective is very important because there are already proton facilities in the US and payers only care about the cost per patient and if proton therapy is cost-effective relative to their own reimbursements. Finally, we only performed analyses among a specific patients’ age cohort, while factors such as age difference, smoking history, breast cancer subtype, and prior heart diseases etc. may have significant impacts on the transition probabilities. These personalized factors will be further investigated by us in a future study.

Multiple studies [39–41] have shown that hypo-fractionated PMRT is safe and effective and can provide similar tumor control rate as standard 25 fractions radiotherapy for carefully selected post-mastectomy patients. It is also much more convenient for the patients and caregivers and can significantly cut treatment costs [41, 42]. The patient cohort we used to derive transition probabilities for advanced PMRT techniques were not selected for hypo-fractionated PMRT in our clinic. The comparison of total costs with and without hypo-fractionation and cost-effectiveness evaluation of various PMRT techniques for hypo-fractionated PMRT patients will be investigated by our group in the near future.

## Conclusions

In summary, most advanced PMRT techniques are more cost-effective than SOC at a WTP threshold of $100,000/QALY, while none of the advanced techniques is more cost-effective than SOC at a WTP threshold of $50,000/QALY. For the 55-year-old patient cohort, IMRT might be a cost-effective option.

## Supplementary information

**Additional file 1: Figure S1.** QALY versus treatment cost for all PMRT techniques. The dots representing techniques that are not dominated by any other technique are joined in this figure, and ICER values are only calculated for these techniques as shown in Table 4. **Figure S2.** Tornado diagrams of one-way analyses results comparing SOC with (a) IMRT, (b) STD-VMAT, (c) NC-VMAT, (d) MA-VMAT, (e) TOMO, (f) MIXED and (g) IMPT. Bars indicate range of costs per QALY for given range-specific model input variables. P_: probability of developing certain radiogenic side effect using certain PMRT technique. **Figure S3.** Cost-effectiveness acceptability curves from PSA that comparing the cost-effectiveness of SOC and (a) IMRT, (b) STD-VMAT, (c) NC-VMAT, (d) MA-VMAT, (e) TOMO, (f) MIXED and (g) IMPT at different willingness to pay (WTP) thresholds. Two dashed lines represent WTP thresholds of $50,000/QALY and $100,000/QALY.

## Data Availability

The dataset used for the analysis in the study can be made available from the corresponding author upon reasonable request.
