# Supplementary material for: Cost-effectiveness analysis of advanced radiotherapy techniques for post-mastectomy breast cancer patients
Source: Cost Eff Resour Alloc. 2020 Aug 3;18:26. doi: 10.1186/s12962-020-00222-y (PMC7398314; doi:10.1186/s12962-020-00222-y)
Supplement: Supplementary file 1 — Additional file 1: Figure S1. QALY versus treatment cost for all PMRT techniques. The dots representing techniques that are not dominated by any other technique are joined in this figure, and ICER values are only calculated for these techniques as shown in Table 4. Figure S2. Tornado diagrams of one-way analyses results comparing SOC with (a) IMRT, (b) STD-VMAT, (c) NC-VMAT, (d) MA-VMAT, (e) TOMO, (f) MIXED and (g) IMPT. Bars indicate range of costs per QALY for given range-specific model input variables. P_: probability of developing certain radiogenic side effect using certain PMRT technique. Figure S3. Cost-effectiveness acceptability curves from PSA that comparing the cost-effectiveness of SOC and (a) IMRT, (b) STD-VMAT, (c) NC-VMAT, (d) MA-VMAT, (e) TOMO, (f) MIXED and (g) IMPT at different willingness to pay (WTP) thresholds. Two dashed lines represent WTP thresholds of $50,000/QALY and $100,000/QALY. [file 12962_2020_222_MOESM1_ESM.docx]

**Additional Materials**

**Figure S1** QALY versus treatment cost for all PMRT techniques. The dots representing techniques that are not dominated by any other technique are joined in this figure, and ICER values are only calculated for these techniques as shown in table 4.


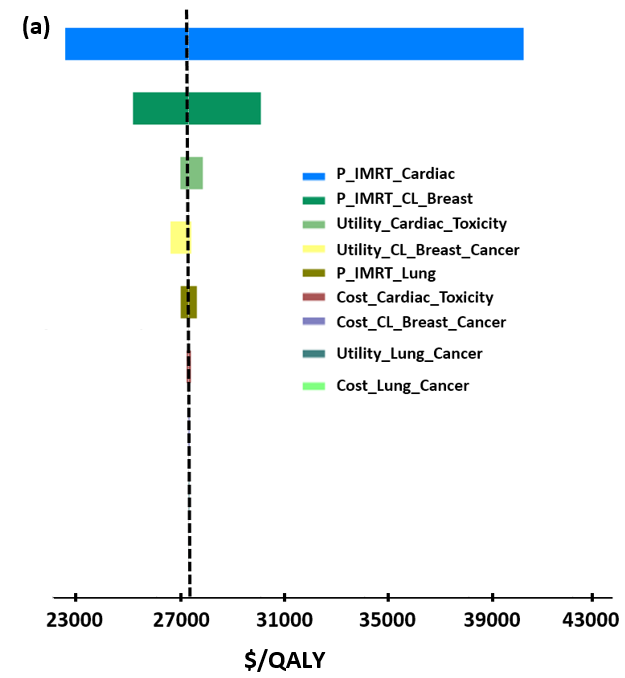


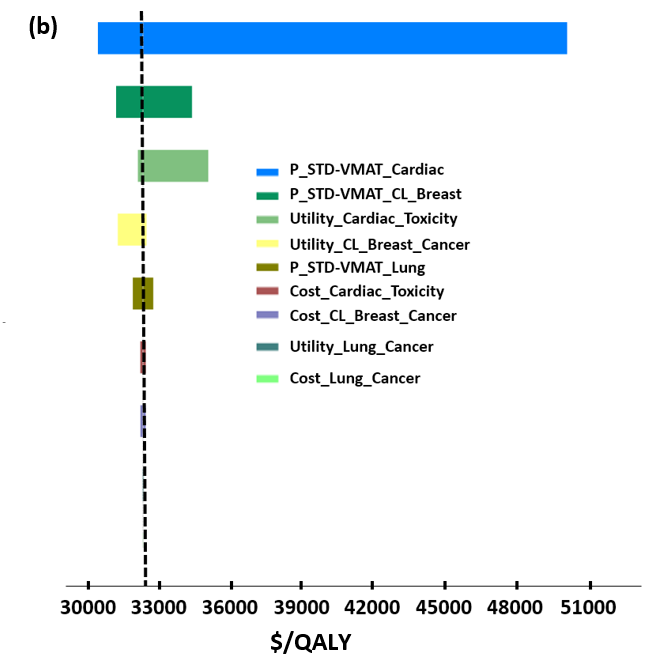


**
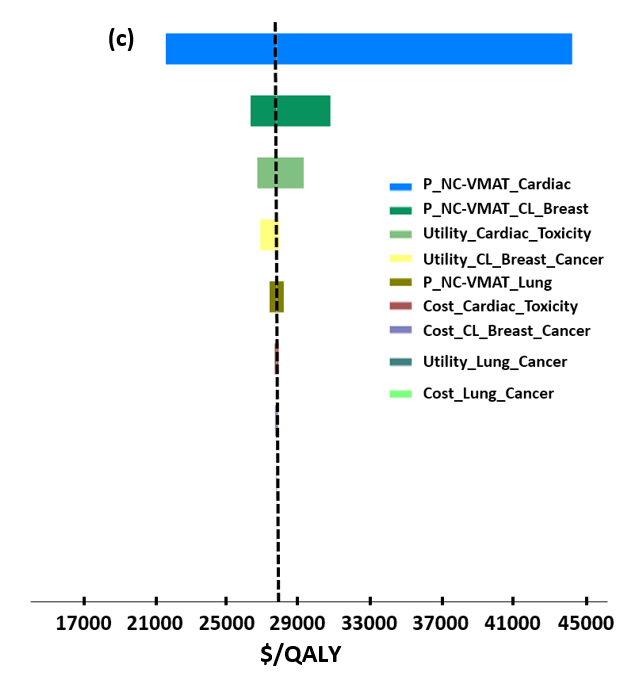
**

**
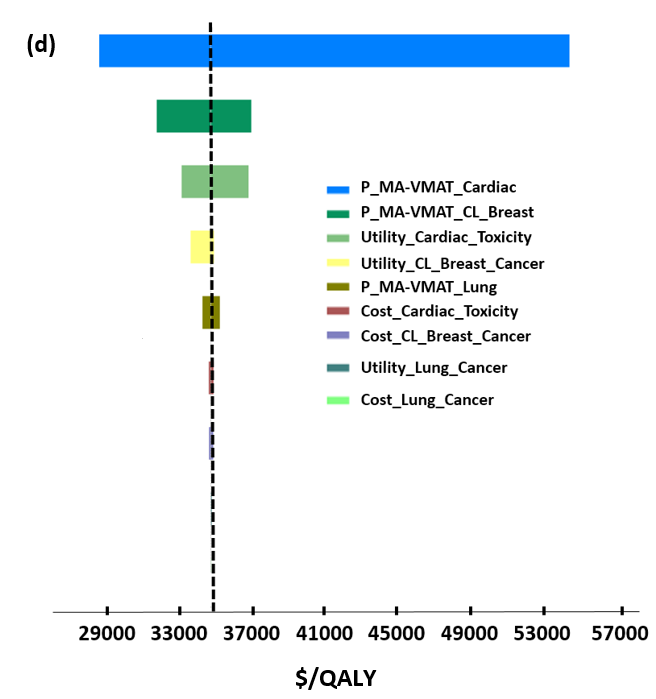
**

**
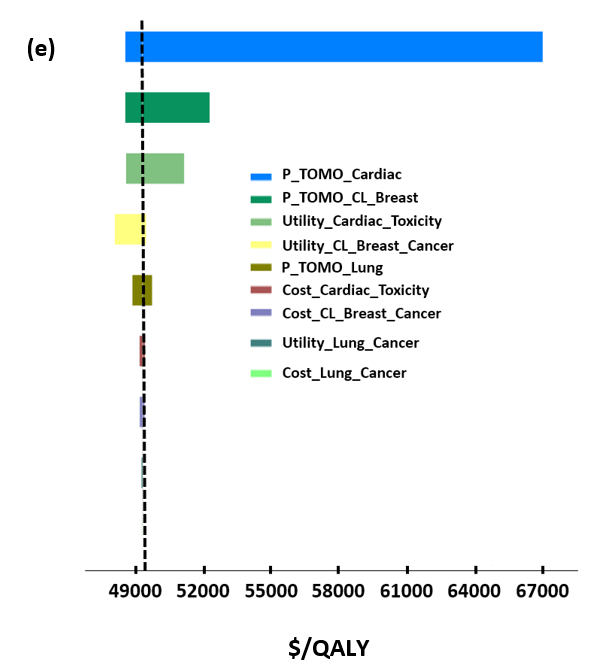
**

**
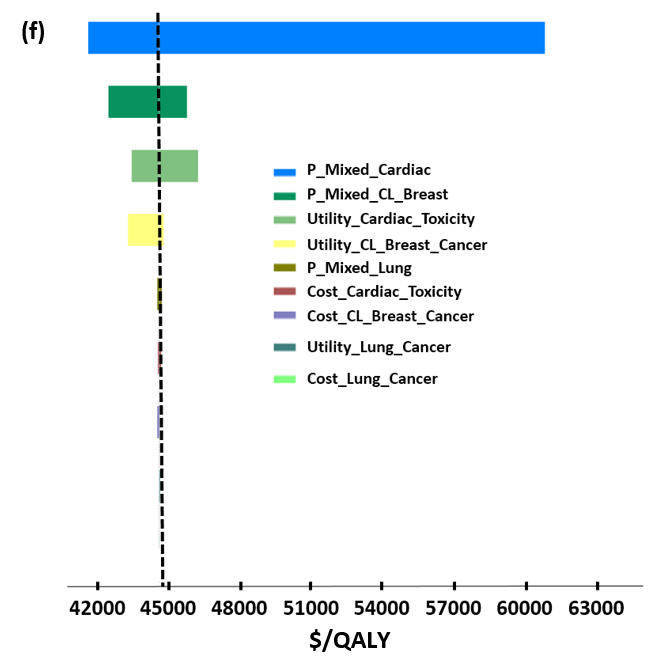
**

**
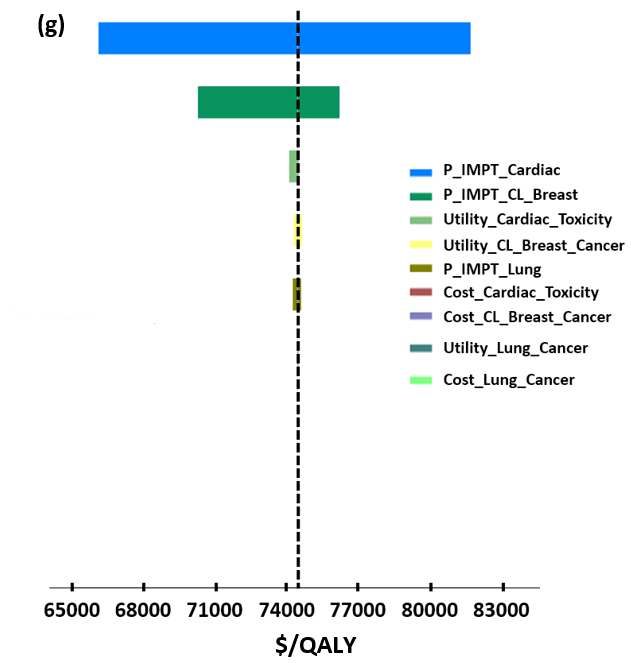
**

**Figure S2** Tornado diagram of one-way analyses results comparing SOC with (a) IMRT, (b) STD-VMAT, (c) NC-VMAT, (d) MA-VMAT, (e) TOMO, (f) MIXED and (g) IMPT. Bars indicate range of costs per QALY for a given range-specific model input variables. P_: probability of developing certain radiogenic side effect using certain PMRT technique.


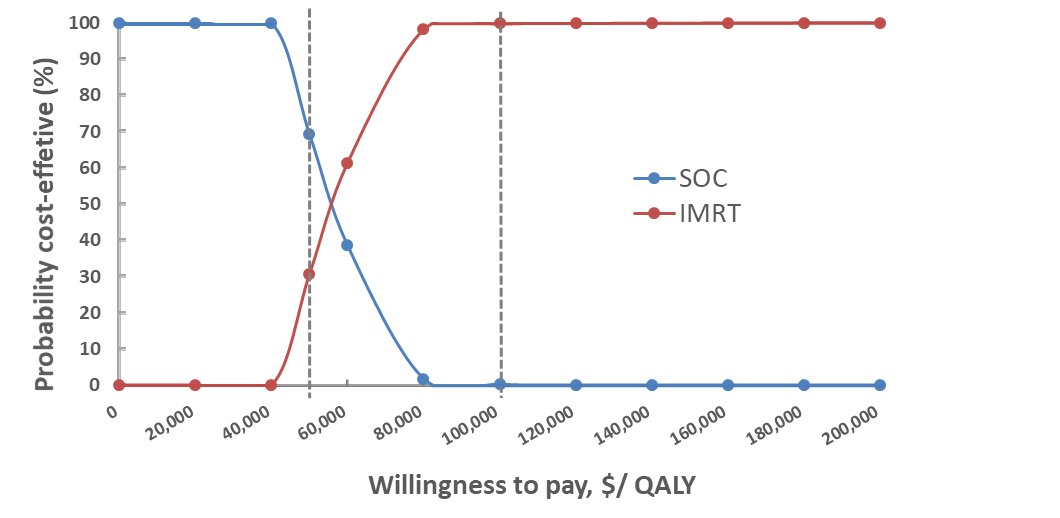


**(a)**


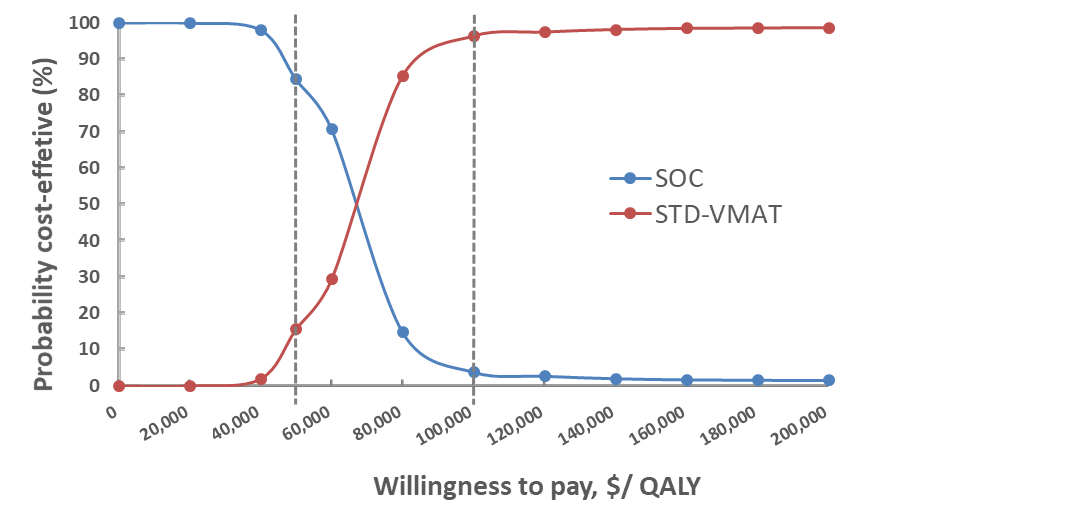


**(b)**


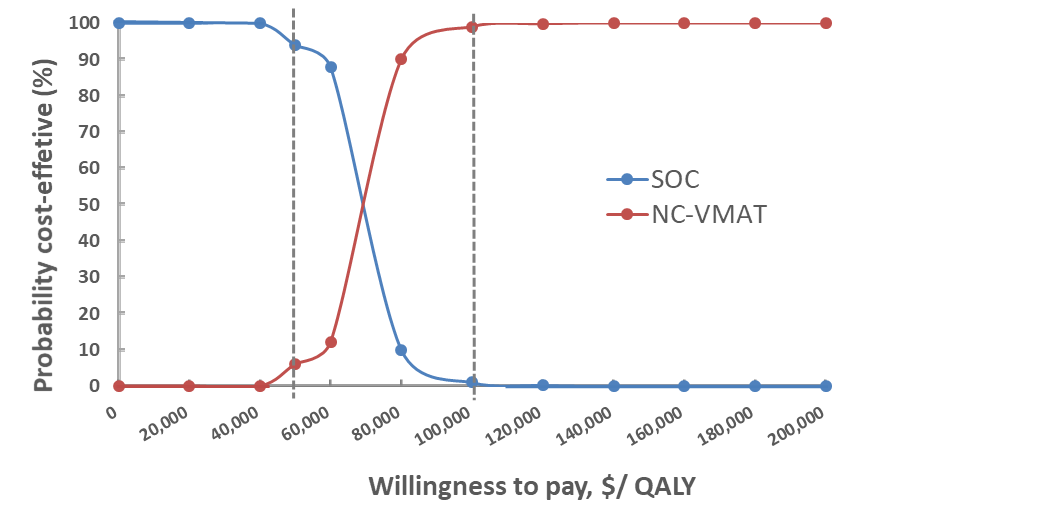


**(c)**


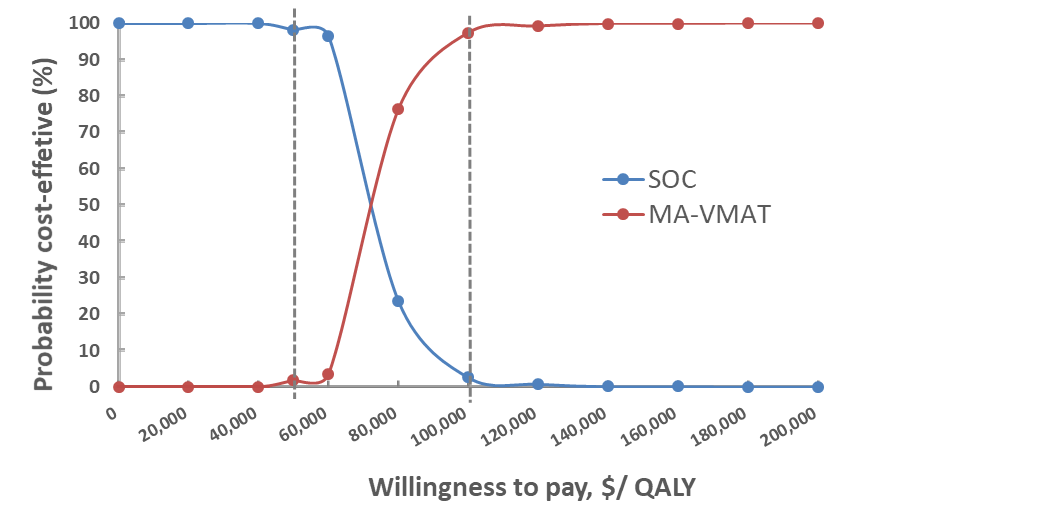


**(d)**


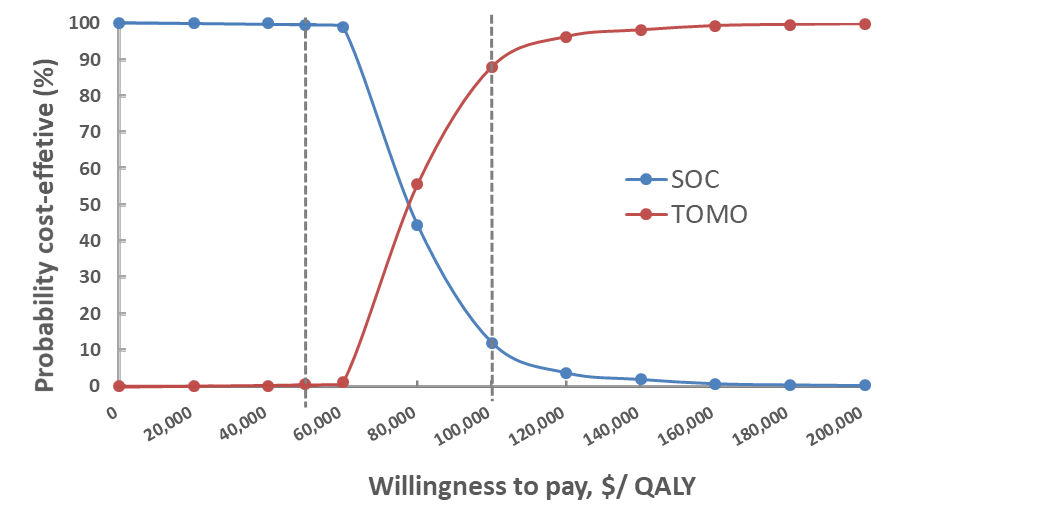


**(e)**


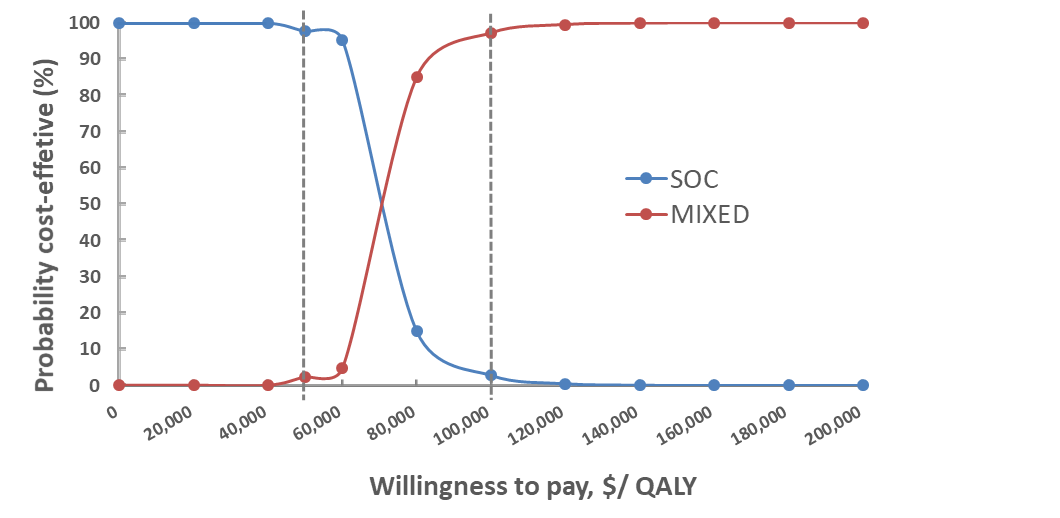


**(f)**


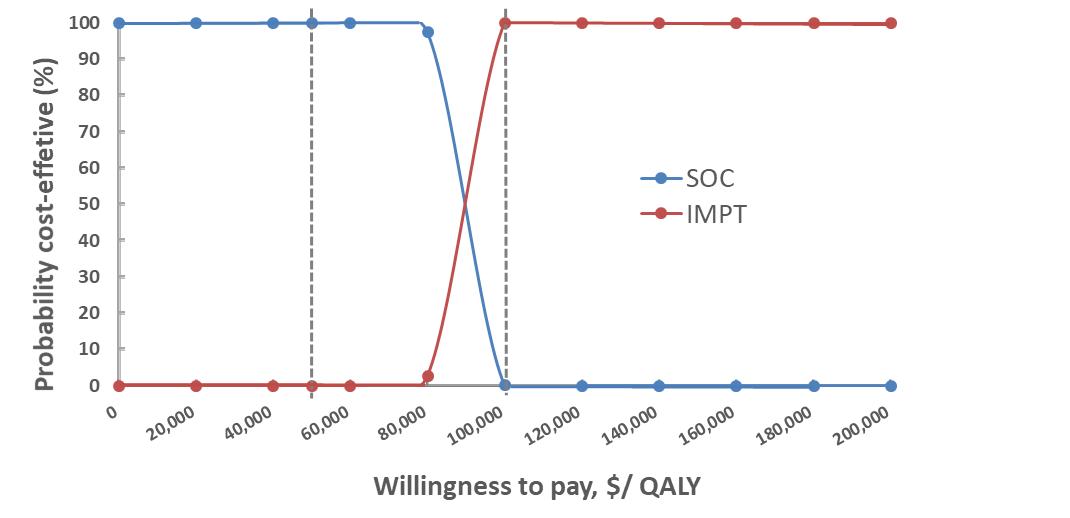


**(g)**

**Figure S3** Cost-effectiveness acceptability curves from PSA that comparing the cost-effectiveness of SOC and (a) IMRT, (b) STD-VMAT, (c) NC-VMAT, (d) MA-VMAT, (e) TOMO, (f) MIXED and (g) IMPT at different willingness to pay (WTP) thresholds. Two dashed lines represent WTP thresholds of 50,000 $/QALY and 100,000 $/QALY.
